# A dyadic stimulus set of audiovisual affective displays for the study of multisensory, emotional, social interactions

**DOI:** 10.3758/s13428-015-0654-4

**Published:** 2015-11-05

**Authors:** Lukasz Piwek, Karin Petrini, Frank Pollick

**Affiliations:** 1 Centre for the Study of Behaviour Change and Influence, University of the West of England, 4D17, Coldharbour Lane, BS16 1QY Bristol, UK; 2 Department of Psychology, University of Bath, Claverton Down, BA2 7AY Bath, UK; 3 School of Psychology, University of Glasgow, 58 Hillhead Street, G12 8QB Glasgow, UK

**Keywords:** Biological motion, Voice dialogue, Point-light, Happy, Angry, Social interaction, Multisensory

## Abstract

We describe the creation of the first multisensory stimulus set that consists of dyadic, emotional, point-light interactions combined with voice dialogues. Our set includes 238 unique clips, which present happy, angry and neutral emotional interactions at low, medium and high levels of emotional intensity between nine different actor dyads. The set was evaluated in a between-design experiment, and was found to be suitable for a broad potential application in the cognitive and neuroscientific study of biological motion and voice, perception of social interactions and multisensory integration. We also detail in this paper a number of supplementary materials, comprising AVI movie files for each interaction, along with text files specifying the three dimensional coordinates of each point-light in each frame of the movie, as well as unprocessed AIFF audio files for each dialogue captured. The full set of stimuli is available to download from: http://motioninsocial.com/stimuli_set/.

**Electronic supplementary material:**

The online version of this article (doi:10.3758/s13428-015-0654-4) contains supplementary material, which is available to authorized users.

## Introduction

Every day we observe social interactions around us, and those social scenes typically comprise complex and emotional situations, which engage multiple senses. In order to empirically study the perception of emotions in such complex conditions, a stimulus set is needed that will be flexible enough to enable us to manipulate visual and auditory cues, but simple enough to reduce the enormous complexity of such social scenes.

In visual domain studies, point-light displays have frequently been used to examine the perception of body movement, mainly because this method allows us to study body movement in isolation from other contextual cues such as clothing, facial expression or body shape (Johansson 1973). A large number of studies have shown that observers can recognise specific actions (Dittrich 1993; Vanrie and Verfaillie 2004), gender (Mather and Murdoch 1994; Troje 2002), age (Montepare and Zebrowitz-McArthur 1988), identity (Cutting and Kozlowski 1977; Hill and Pollick 2000) and affect (Dittrich et al. 1996; Pollick et al. 2001; Atkinson et al. 2004; Clarke et al. 2005) from just a set of point-lights representing the main joints of human movement. In comparison with solid-body displays, point-light displays have the advantage of being much easier to manipulate and adapt to a variety of studies (Hill et al. 2003). However, only a limited number of studies have utilised point-light displays in the context of emotional social interactions. One prominent example is the motion capture database by Manera et al. (2010) which included 20 communicative interactions such as sharing, ordering, giving information, helping and offering, but used a small number of actors, without voice capture and with limited inclusion of emotional valence. Another relevant stimulus set was developed by Busso et al. (2008), who created a motion capture database of actors’ faces and hands combined with conversational speech. The authors recorded a number of improvised interactive scenarios, with the emotional interactions representing happiness, anger, sadness, frustration and a neutral emotional state. However, Busso et al. (2008) stimuli set only shows actors’ heads and hands, which makes it unsuitable for examining perception in a multiagent communication. Clarke et al. (2005) describe their attempt to use dyadic point-light displays to examine the perception of a broad range of emotions: anger, love, sadness, fear and joy. The authors used emotional point-light interactions which were captured during scripted dialogues. However the auditory dialogues were not included with the visual displays, and their stimulus set has not been made publicly available.

Studies in the auditory domain have utilised a number of speech-oriented stimulus sets that use naturalistic, interactive discourse and represent a varied range of emotional interactions, some with video recordings. Douglas-Cowie et al. (2003) created the Belfast Natural Database, which comprises 125 natural clips taken from television shows. Scherer and Ceschi (1997) conducted the Geneva Airport Lost Luggage study using 109 natural, unobtrusive video tapings of passengers at a lost luggage counter, followed by interviews with the passengers. There also exists a Reading-Leeds database with five hours of material recorded from natural, unscripted interviews taken from radio and television shows, in which speakers were induced by interviewers to relive emotionally intense experiences (Roach et al. 1998). However despite the obvious benefits of naturalistic stimuli (most critically, their unrehearsed and unscripted nature), in many cases they are not appropriate for the intended purpose of controlled psychophysical studies, due to difficulty in controlling factors such as the language spoken, the length of clips, and the ambiguous specification of emotional expressions (Douglas-Cowie et al. 2003; Ververidis and Kotropoulos 2006). Finally, a number of affective auditory stimulus sets have been created in controlled conditions. For instance, the Montreal Affective Voices set consists of 90 nonverbal affect bursts corresponding to a range of emotions, recorded from ten different actors (Belin et al. 2008). Another stimulus set, International Affective Digitized Sounds, consists of vocal and nonvocal sounds mixed with pleasant and unpleasant auditory stimuli (Bradley and Lang 1999). However, similarly to the issues highlighted with visual-only point-light sets described above, the speech-oriented stimulus sets would also be difficult to use in studies examining multisensory perception of emotional social interactions.

In spite of the broad range of affective stimulus sets available in both the visual and auditory domains, none of the existing sets combine body movement and voice in the context of emotional social interactions. This is surprising, considering that everyday social scenes are typically multi-agent events which engage multiple senses. To address this research gap, we created a set of dyadic, emotional, social interactions stimuli. Our set consists of 238 unique clips that present happy, angry and neutral emotional interactions with low, medium and high levels of emotional intensity. The set was derived from 756 motion and voice captures from nine different couples. This set was then evaluated in a between-design experiment.

It is difficult to obtain realistic emotional interactions for the entire spectrum of emotions using simulated actions, and so we decided to capture only happy and angry interactions with different levels of intensity. In normal daily life, people express emotions with varying intensities, and we wanted to take this variability into consideration. We also wanted to obtain a large variance of interactions within the happy and angry emotional expressions rather than having a broad scope of different emotions. Furthermore, anger and happiness are the most frequently reported emotions when people are asked to introspect about their experienced affects (Scherer and Tannenbaum 1986). Both of these emotions represent emotional states or moods that might last for an extended period of time (Ma et al. 2006). Additionally, a number of studies found that actors find angry and happy emotional expressions easy to convey in various scenarios, and observers can easily recognise such expressions (Pollick et al. 2001; Pollick et al. 2002; Ma et al. 2006). We decided to avoid reactive emotions such as surprise or disgust because they are associated with specific movements and are difficult to perform (Konijn 2000; Kleinsmith and Bianchi-Berthouze 2013).

In the following paragraphs we describe the details of the motion and voice recording systems, the capture sessions, and the post-processing of recorded data. We then describe how the final stimulus set was created and validated.

## Stimulus creation

### Motion and voice capture setup and calibration

A group of 20 actors was selected and combined into ten pairs: five experienced (at least five years of acting experience) and five non-experienced (no acting experience) pairs. The mean duration of acting experience for experienced actors was 9.68 years, ranging from 5 to 25 years, and they all reported to have practised improvisation as an essential part of their acting training. Ma et al. (2006) and Rose and Clarke (2009) argued that experienced actors tend to systematically exaggerate emotional expressions, a trait which emerges from their theatrical training. Roether et al. (2009) found no differences between experienced and inexperienced actors in terms of acting quality. Still, Ma et al. (2006) highlighted that exaggerated behaviour could be a part of natural expression and it is sometimes difficult to draw the line between genuine expression and exaggeration. However, Busso et al. (2008) argued that experienced actors typically perform better than inexperienced actors during scripted scenarios. We used both experienced and inexperienced actors in order to address some of the ambiguities in the existing studies regarding the actors’ experience.

All the actors were English-speaking, UK-born males, with a mean age of 26.12 years, ranging from 17 to 43 years. Our goal was to capture wider interpersonal variance in emotional actions, rather than to explore inter-gender effects, and so we recorded only male dyadic interactions. Two actors participated in every session, they knew each other moderately well (e.g., they were colleagues but not partners) and they were paid for their time. Before each session the actors were briefed on the purpose of the study and signed a consent form.

**Fig. 1:**
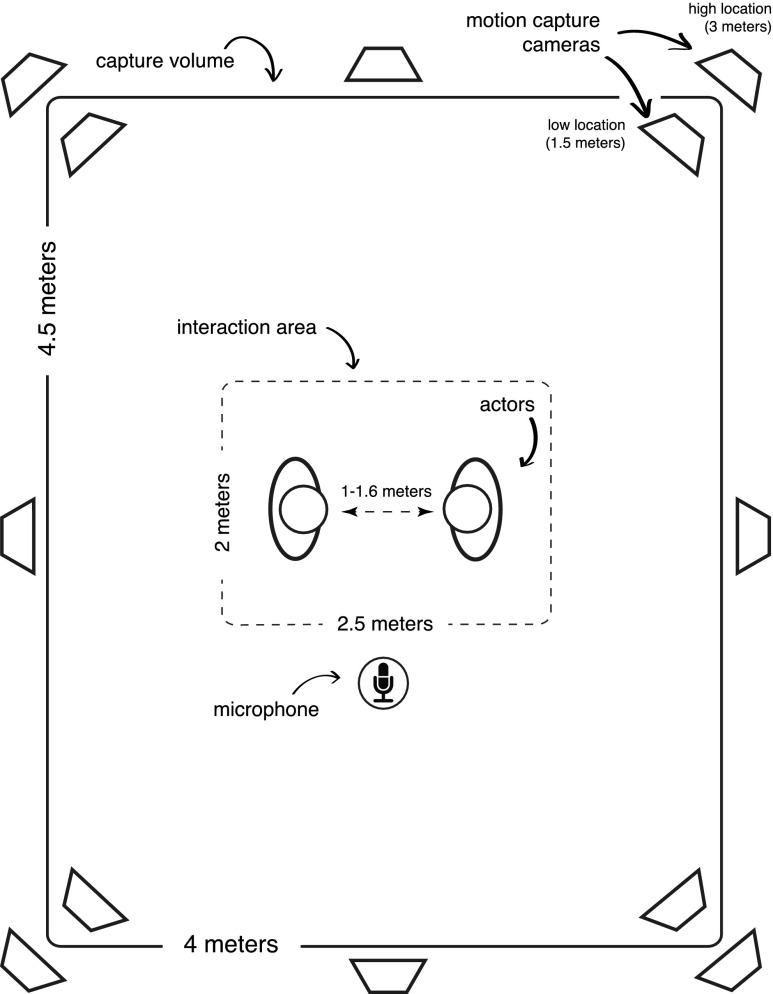
Motion capture room - cameras, microphone setup and capture area (schematic view from the top)

Motion capture took place at the University of Glasgow in the School of Psychology, using 12 Vicon MXF40 cameras (Vicon, 2010) which offer online monitoring of 3D motion signals. At all times, the system was recording at a rate of 120 frames per second (fps). The audio capture used a Tescam HD-P2 two-channel digital audio recorder connected to an AKG D7S Supercardioid Dynamic Microphone, and it recorded at 44.1kHz with a 24-bit sampling rate. During the recording, the audio capture was fully synchronised with the motion capture via the Vicon Analogue Card (Vicon, 2010). The entire capture setup, including floor measurements and the location of cameras, microphone and actors, is illustrated in Fig. 1. Vicon Nexus 1.3 (Vicon, 2010) was used for most of the capture operations including the calibration, capturing, storage, and post-processing of raw capture data.

**Fig. 2:**
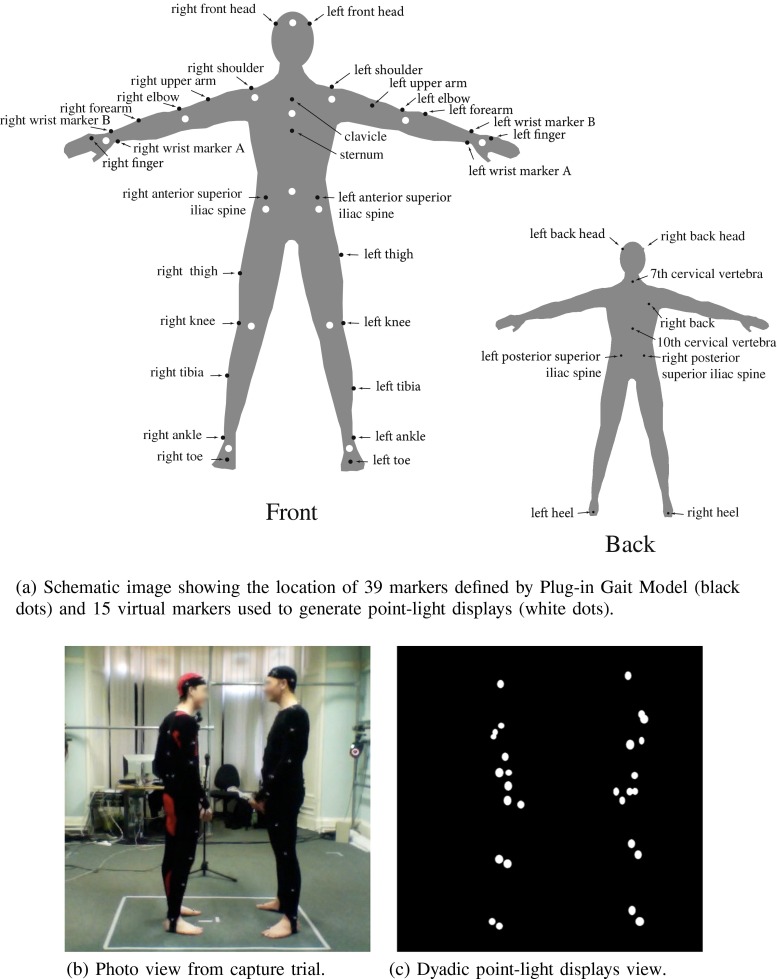
Images illustrating various stages of motion capture including (**a**) Plug-in Gait model and virtual marker location, (**b**) photo of actors from capture session and (**c**) dyadic point-light displays

After calibration of the motion capture system, each capture session started with the taking of actors’ measurements and the placement of 39 retroreflective, 14mm, spherical markers on specific anatomic locations on their bodies. These anatomical locations were defined by the Plug-in Gait Model (black dots on Fig. 2a) which is based on the widely accepted Newington-Helen Hayes gait model. It uses a defined marker set and a set of subject measurements to create outputs of the joint kinematics and kinetics for each gait analysis participant (Kadaba et al. 1990; Davis et al. 1991). Supplementary Table 3 describes the exact anatomical locations of the markers.

During the capture session actors were positioned, one facing the other, at a distance specified by a marked position on the floor, approximately 1.3 metres. This interpersonal distance varied between 1 - 1.6 metres (Fig. 1) and it flexibly changed during the capture trials, depending on how much actors moved when interacting. At the beginning of each single capture trial actors were asked to come back to the start position marked on the floor. The overall space of interaction was limited to around 2.5 x 2 metres (Fig. 2b), but since the participants were within the comfortable personal space as defined by Hall (1966), we expected that their natural interaction would not be affected by proxemics.

We captured three types of emotional interaction: angry, happy and neutral. Angry and happy interactions were captured at three different intensity levels: low, medium and high. Actors were given relative freedom in expressing the emotions during interactions (Rose and Clarke 2009). They were encouraged to act naturally, but they were instructed to avoid touching each other and we were careful to give them only verbal instructions rather than performing actions ourselves (Clarke et al. 2005; Ma et al. 2006; Roether et al. 2009). People typically use touch to share their feelings with others, and to enhance the meaning of other forms of verbal and non-verbal communication (Gallace and Spence 2010). Touch also appears very early in human development and naturally becomes on its own a powerful indicator of affect (Harlow 1958).

To help the actors convey angry and happy emotions at different levels of intensity, they were given short and simple emotional scenarios and asked to imagine themselves in those situations. Supplementary Table 1 describes the exact scenarios given to actors. The order of scenarios given and the order of emotions to be conveyed was randomised for each pair. Actors were also instructed to recall any past situations that they might have associated with the relevant emotional scenario to help them induce the emotion. The hypothetical scenarios were based on simple common situations (Scherer and Tannenbaum 1986). The neutral condition served as a control, and here actors were asked to interact in a neutral, emotion-less manner. In all other conditions, the actors received a verbal explanation of what emotion they should play in a specific scenario. We took care to avoid using any symbolic gestures or other non-verbal suggestions. All actors had a short practice time of up to one minute (if required) to refine their actions before each recording.

Actors were always asked to use the same dialogues when interacting in each single capture trial (i.e. happy, angry or neutral in all intensity levels), with the dialogues being either inquiry (question and answer; actor 1: “Where have you been?”; actor 2: “I have just met with John”) or deliberation (two affirmative sentences; actor 1: “I want to meet with John”; actor 2: “I will speak to him tomorrow”). We purposely chose inquiry and deliberation as the two formats of dialogue, as specified in Krabbe and Walton (1995), because we wanted to ascertain whether those different formats of dialogue influenced the identification of emotional interaction between the actors by the observers. We also picked relatively neutral words for the dialogues, so that they were easy to articulate in either a happy or angry emotional manner.

Each single capture trial lasted no longer than 610 seconds. In each trial, the recording started around 1 second before actors were given a signal to begin the interaction. To signal the start of each capture trial, a 1-second long digital square wave sound was played. Recording stopped around 23 seconds after the actors stopped their interaction. For each pair of actors we completed 10 practice trials before the capture trials. Practice trials were included to give actors more time to prepare, to adjust to their roles and for us to check if the motion and voice capture system had been calibrated correctly. Immediately after the practice trials we initiated the capture trials, during which we collected the material used for creating the stimulus set. For each actor pair we obtained 84 capture trials. These comprised 2 emotions (happy, angry), 3 intensities (low, medium, high), 2 dialogue versions (inquiry, deliberation), 2 actors order, 3 repetitions plus 12 neutral conditions (2 dialogue versions * 6 repetitions of each action). This resulted in a total of 756 film clips for all nine couples. There were another 100 data trials from 10 practice captures for each couple, but these practice captures were excluded from further post-processing.

### Post-processing procedure

There were five main stages of post-processing: (1) calculating the 3D position data from 2D camera data; (2) automatically labelling the reconstructed markers based on the Plug-in Gait model; (3) automatically interpolating missing data points; (4) exporting raw coordinates and creating point-light displays in MATLAB 2010 (Mathworks, 2010), and (5) exporting raw audio dialogues in order to process them in Adobe Audition. The first three operations were executed automatically in Vicon Nexus 1.3 (Vicon, 2010). Creating final point-light displays required a few additional steps. From the trajectories of the 39 original markers, we computed the location of ‘virtual’ markers positioned at major joints of the body. The 15 virtual markers used for all the subsequent computations were located at the joints of the ankles, the knees, the hips, the wrists, the elbows, the shoulders, at the centre of the pelvis, on the sternum, and in the centre of the head (white dots on Fig. 2a). Commercially available software Vicon BodyBuilder (Vicon, 2010) for biomechanical modelling was used to achieve the respective computations. A similar approach has been used in the past by Dekeyser et al. (2002), Troje (2002) and Ma et al. (2006). The advantage of this procedure was that it was a quick and automated way of creating the virtual joint centres for both actors without the need for manual adjustments (Dekeyser et al. 2002; Ma et al. 2006).

After attaching virtual markers, the 3D (x, y, z) position coordinates for those markers were exported from Vicon Nexus 1.3 (Vicon, 2010) as a tab-delimited text file. Those coordinate files were formatted in such a way that position coordinates were represented in columns, while each frame of data record was represented in rows. Coordinate files were imported into MATLAB 2010 (Mathworks, 2010) and an algorithm was applied to generate the final point-light displays. The algorithm was based on that used by Pollick et al. (2001) which converted 15 virtual markers from each actor into point-light displays, generated as white dots on a black background from the side view, as seen in Fig. 2c. The algorithm exported point-light displays in the Audio Video Interleave (AVI) format, with a frame size of 800 by 600 pixels. The frame rate of exported displays was reduced from the original 120 fps to 60 fps, because MATLAB 2010 (Mathworks, 2010) and Adobe Premiere 1.5 (Adobe Systems, 2004), which were used for creating the final displays, only allowed editing of the movie up to 60 fps.

The audio dialogues recorded with the Vicon Analogue Card were all saved by Vicon Nexus 1.3 (Vicon, 2010) in the Audio Interchange File Format (AIFF), and each audio dialogue was automatically linked with the corresponding capture trial. Adobe Audition 3 (Adobe Systems, 2007) was used to post-process the dialogues. Every audio dialogue was first amplified by 10dB and then a noise reduction was applied. Following this all audio dialogues were normalised to create a consistent level of amplitude, and to obtain the average volume of around 60dB. Finally, each audio dialogue was exported as a Waveform Audio File Format (WAV) file with a resolution of 44.1kHz and 24-bit sampling rate.

### Creation of final stimulus set

Adobe Premiere 1.5 (Adobe Systems, 2004) was used to create a final stimulus set. The AVI point-light displays produced by MATLAB 2010 (Mathworks, 2010) were imported into Adobe Premier 1.5 (Adobe Systems, 2004) together with the corresponding WAV dialogues post processed with Adobe Audition 3 (Adobe Systems, 2007). Initially, each point-light display was combined with its corresponding WAV dialogue. The initial recording of the interaction was signalled by a sound (one second long, square-wave buzzer signal). The end of the recording occurred 500 ms after the end of the actors dialogue. The length of the final, truncated display varied between 2.5 and 4.5 seconds. Thus the edited clip represented the original interaction of the actors in its entirety after shortening the initial and final segments where nothing occurred. The eliminated segments before and after the selected clip were initially recorded only to enable us to obtain longer ‘technical margins’ for post-processing the length of final displays.

All displays with truncated start/end points were exported to AVI format in three versions: auditory-only (dialogues), visual-only (point-light displays) and audio-visual (dialogues combined with point-light displays). The final, non-validated stimulus set was composed of 238^1^ unique displays which consisted of: 9 actor couples, 2 emotions (happy and angry), 3 intensities (low, medium, high), 2 dialogue versions (inquiry, deliberation), 2 repetitions plus 26 neutral displays. However, each display was created in three modality formats: visual point-light displays, auditory dialogues and a combination of point-light displays and dialogues. Therefore the final count of all displays in stimulus set with three modality formats was 714.

The stimulus set (Supplementary Material 2) can be downloaded from the following sources: http://motioninsocial.com/stimuli_set/. The stimulus set is organised into nine folders, with each folder being labelled with a letter representing a different actor couple. Within each folder, every single display is represented by five files:

- three AVI files in audio-visual, auditory, and visual versions;
- one TXT file with unprocessed motion capture coordinates for the corresponding display (Supplementary Material 1 in the Appendix includes *R* routine with exact description of what each column stands for);
- one WAV file with unprocessed dialogue capture for the corresponding display.

Supplementary Table 3 includes detailed characteristics for each display. This table is also available as *Microsoft Excel* XLS file together with Supplementary Material 2 for easier browsing.

It is worth noting that the reason why we created only 238 unique clips from 756 original captures was due to the technical quality of the motion and voice captures, and the quality of the acting. The most common issues that occurred during motion capture were: marker occlusion during the capture, distortion of audio or visual noise from ambient light in the capture volume, and errors made in dialogue by actors. Those issues lowered the quality of the displays or made their further processing impossible for the final stimuli set. We were aware prior to the experiment that such issues might occur, hence we recorded the same interactions six times to maximise the number of usable high quality displays.

## Collection of normative data

### Methods

We conducted a series of between-subject experiments to examine how accurately the emotional interactions were identified by observers when presented with the displays as point-lights (visual group), voice dialogues (auditory group) or a combination of point-lights and dialogues (audio-visual group). The reason for using a between-subject design was to avoid audio-visual facilitation, or carry-over effects, which could impact emotional identification when visual, auditory and audio-visual displays are presented together in one set. Audio-visual facilitation has been demonstrated in studies using emotional faces and voices, when audio-visual conditions enhanced perceived emotion in comparison with auditory-only and visual-only conditions (Collignon et al. 2008; Piwek et al. 2015). We also wanted to restrict the presentation of every display to a single occasion to avoid the practice effects that can occur when participants see a repetition of a specific stimulus (Heiman 2002).

We separately recruited a total of 43 participants for three independent groups: a visual group (15 participants, 7 of them male, with a mean age of 25.8 years, ranging from 17 to 45 years), an auditory group (13 participants, 6 of them male, with a mean age of 20.5 years, ranging from 17 to 26 years), and an audio-visual group (15 participants, 8 of them male, with a mean age of 22.5 years, ranging from 18 to 37 years). All participants were English-speaking and UK-born, and they all reported normal hearing and normal or corrected-to-normal vision. All the participants were naive to the purpose of the study and they lacked any prior experience with point-light display movies or images. The study received ethical approval from the University of Glasgow’s Faculty of Information and Mathematical Sciences Ethics Review Board. Every participant signed a consent form and was paid for his/her time.

We used the stimulus set described in Section “Creation of final stimulus set”, composed of happy, angry and neutral dyadic interactions presented as point-light displays (visual group), voice dialogues (auditory group) and a combination of point-light displays and voice dialogues (audio-visual group). The task was exactly the same for all three groups. After being presented with the display, participants were given two questions. First, participants were asked to identify whether interaction was happy or angry. They did so by choosing ‘H’ for happy or ‘A’ for angry on the keyboard. Immediately after their response, the second screen was presented. In this second screen participants were asked how confident they were about their choice of emotion on a rating scale from *1* to *9*, where *1* referred to *very low confidence* and *9* referred to *very high*. Each display was presented only once and the order of all displays was randomised. We used Neurobehavioral Presentation 13.1 software (Neurobehavioral Systems, 2008) to present the displays and collect the responses.

### Results

**Fig. 3:**
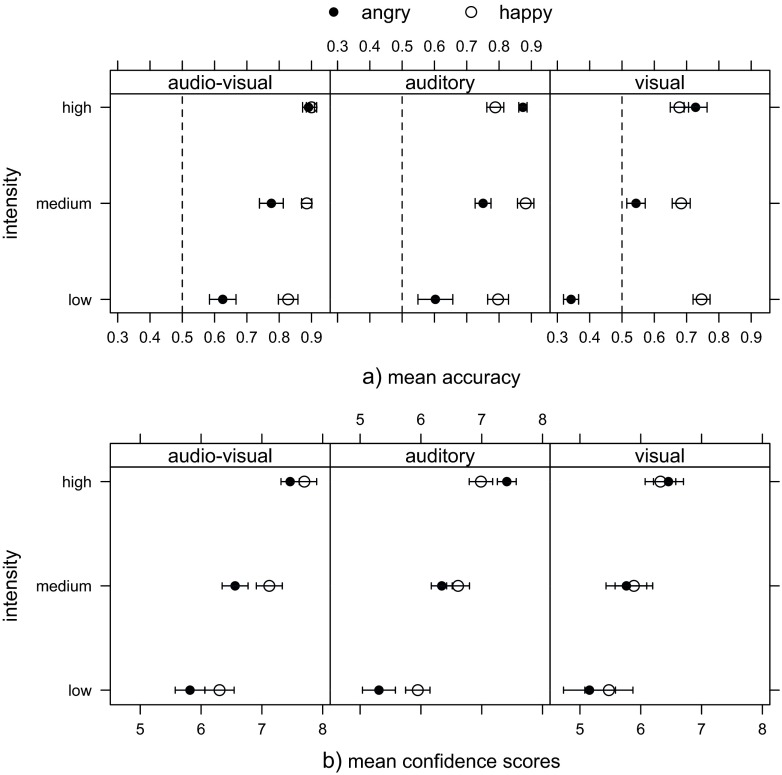
Mean (**a**) identification accuracy and (**b**) confidence rating of emotion judgments for happy and angry displays at low, medium and high intensity in visual, auditory and audio-visual experiment. The error bars represent one standard error of the mean, and the dashed line indicates the level of chance (0.5)

Figure 3 shows accuracy and confidence ratings for each group, averaged across happy and angry displays on low, medium and high intensity levels. There was a broad variance in participants’ responses. We conducted two separate mixed design ANOVAs on an averaged number of correct responses, and averaged confidence ratings, with ‘emotion’ (happy and angry) and ‘intensity’ (low, medium, high) as within-subject factors, and ‘group’ (visual, auditory, audio-visual) as a between-subject factor. To summarise the key results, we found that observers in the audio-visual and auditory groups showed better accuracy of emotion identification (F(2,40) = 93.01, p < 0.001, ${\eta _{G}^{2}}$ = 0.40) and higher confidence (F(2,40) = 4.42, p < 0.05, ${\eta _{G}^{2}}$ = 0.16) in their judgements than those in the visual group (Fig. 3a). Overall, happy displays were identified more accurately than angry displays (F(1,40) = 17.86, p < 0.001, ${\eta _{G}^{2}}$ = 0.17), but emotional intensity played a key role in identification accuracy and confidence of responses. Specifically, accuracy increased with higher levels of intensity for angry displays, but intensity did not influence accuracy of judgements for happy displays (F(2,80) = 36.89, p < 0.001, ${\eta _{G}^{2}}$ = 0.21); Fig. 3b. However, in all groups confidence increased with higher intensity displays (F(2,80) = 34.51, p < 0.001, ${\eta _{G}^{2}}$ = 0.07).

**Fig. 4:**
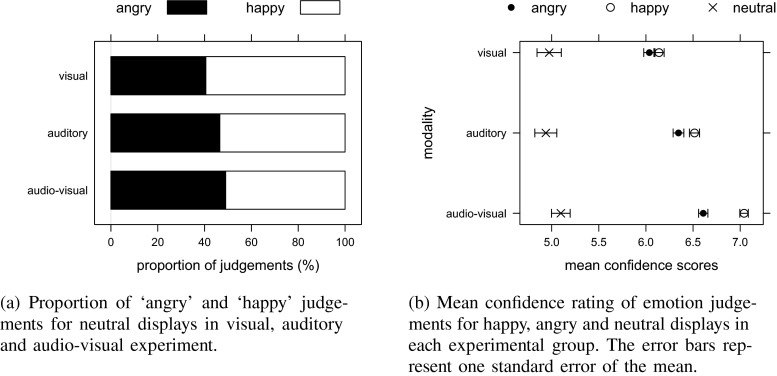
Results for neutral displays from (**a**) proportion of ‘angry’/‘happy’ judgements and (**b**) confidence ratings. There was no specific bias to judge neutral display as either ‘happy’ or ‘angry’, and participants were less confident in their judgements when rating neutral rather than emotional displays

We also wanted to establish whether the proportion of angry judgements was different from the proportion of happy judgements when participants viewed neutral displays. The binomial test showed there was no significant difference in the proportion of happy and angry judgements for neutral displays in the auditory (p = 0.17), visual (p = 0.11) and audio-visual (p = 0.72) groups, as seen on Fig. 4a. In addition, we compared the average confidence ratings given for neutral, happy, and angry displays in each group. A mixed design ANOVA on averaged confidence ratings, with ‘emotion’ (happy and angry) as a within-subject factor and ‘group’ (visual, auditory, audio-visual) as a between-subject factor, showed a significant effect of ’emotion’ (F(2,76) = 117.82, p < 0.001, ${\eta _{G}^{2}}$ = 0.30), but no effect of ’modality’ (F(2,38) = 0.99, p = 0.38, ${\eta _{G}^{2}}$ = 0.04), and no interaction between ’emotion’ and ’modality’ (F(4,76) = 2.23, p = 0.07, ${\eta _{G}^{2}}$ = 0.02). Indeed, pairwise comparison showed that neutral displays were rated with lower confidence than both happy and angry displays (p < 0.001), as seen in Fig. 4b.

In a series of Welsch t-tests we also examined if there was any difference between male and female participants in their emotional recognition accuracy and confidence ratings. The results demonstrated no significant differences between genders in the audio-visual group (accuracy [t = -0.55, df = 79.78, p = 0.58]; confidence [t = -1.4, df = 87.41, p = 0.16]), the auditory group (accuracy [t = 1.277, df = 57, p = 0.21]; confidence [t = 0.04, df = 64.94, p = 0.97]) or the visual group (accuracy [t = -0.36, df = 86.12, p = 0.72]; confidence [t = 2.17, df = 64.25, p = 0.07]).

In the Methods Section “Motion and voice capture setup and calibration” we explained that we used two groups of actors to record our stimulus set: experienced (at least five years of actor training) or non-experienced (no experience in acting). We wanted to examine whether actors’ level of experience impacted how accurately participants were able to identify the emotions portrayed by those actors. We carried out a one-way ANOVA on the average number of correct emotional responses obtained for happy and angry displays, with ‘actors experience’ (experienced actors, non-experienced actors) as a within-subject factor, and found a weak significant effect of this factor (F(1,44) = 4.53, p = 0.04, ${\eta _{G}^{2}}$ = 0.01). In fact, emotions portrayed by non-experienced actors were judged slightly more accurately (*M* = 0.75) than those portrayed by experienced actors (*M* = 0.72), but the scale of difference was almost negligible. Finally, we carried out a one-way ANOVA to examine whether there was any effect of ‘dialogue type’ (inquiry or deliberation) on the accuracy of judgements, but found no significant effect (F(1,44) = 0.01, p = 0.92, ${\eta _{G}^{2}}$ = 0).

Supplementary Table 3 shows the identification accuracy and confidence rating averaged across participants in each group for every display. Supplementary Table 3 is also available as an XLS file in the Supplementary Materials to enable easier searching for most/least accurately identified displays, or to sort by specific conditions for easier browsing.

## Discussion

In this paper we describe the development of the first data set that is able to be used for the study of audio-visual integration from emotional social interactions. Using a passive optical motion capture system, synchronised with audio capture, we recorded 756 interactions between nine different pairs of actors. Captured movement and conversations were converted into formats that were useful for animation as point-light displays combined with voices. The final stimulus set consists of 238 unique clips that demonstrate happy, angry and neutral emotional interactions with low, medium and high levels of emotional intensity. The set has been evaluated in a normative empirical study, as described above.

There are three main features that make our stimulus set particularly suitable for the study of audio-visual integration in the social context. First, we captured both movement and voice in a synchronised manner, and therefore provide the first data set to study audio-visual emotional interactions. Stimulus sets with point-light dyadic interaction have been created before (Manera et al. 2010) but none have combined point-light displays with dialogue. This stimulus set has been designed to study point-light display and voice as combined stimuli or separate stimuli if such an approach is required. The stimuli also consist of entire body movements, in contrast with other existing stimulus sets, which include only discrete parts of the body (e.g. only faces and hands in Busso et al. (2008)).

Second, we simplified the design of the emotional component of the stimulus set by using only happy and angry emotional interactions. Existing stimulus sets include a broader spectrum of emotional expressions (e.g. Busso et al. (2008)), but many of those expressions are difficult to validate considering the ambiguous and reactive nature of some emotions, such as fear or disgust (Ma et al. 2006; Roether et al. 2009). Differences between the perception of happy and angry interactions have been widely reported in the neuroimaging and multisensory literature (e.g. Massaro and Egan, 1996; Fox et al, 2000; Ikeda and Watanabe, 2009). From a practical perspective, it was also easier for our actors to perform happy and angry interactions and for us to create scenarios to help them demonstrate these emotions. This also enabled us to increase variability within the emotional expressions of anger and happiness, by capturing interactions at three different levels of intensity. During the capture of the stimulus set, we used realistic scenarios and role-plays to make the stimulus set more ecologically valid and to help the actors engage in the scene in a more realistic way (Risko et al. 2012). We also used a mix of experienced and inexperienced actors during the recording to increase the variety that might have arisen from any acting strategies that people used to express emotions (Ma et al. 2006).

Third, different parameters of dyadic point-lights are easily customisable due to the universal format in which we have made these data available - as a set of 3D coordinates organised in time-series tables within tab-delimitated text files. Actors’ motion can be analysed, extracted and manipulated. Single actor displays can be easily created, and parameters such as orientation, speed or size of point-lights can easily be changed. Audio dialogues were also normalised and are provided in a widely available WAV format so they can be easily manipulated and analysed for any speech-related cues.

In terms of practical applications, our stimuli set with normative validation has already been successfully used in a study by Piwek et al. (2015), who examined if the audiovisual facilitation of emotion recognition previously found in simpler social situations extends to more complex and ecological situations. The authors selected only a small subset of the described stimuli set, using eight angry and eight happy displays that were identified with at least 75 % accuracy. An additional auditory condition was also introduced where voice dialogues were filtered with brown noise or a low-pass filter in order to decrease the reliability of the auditory signal. In the first experiment, participants were presented with visual, auditory, auditory filtered/noisy, and audiovisual congruent and incongruent clips. Piwek et al. (2015) asked participants to judge whether the two agents were interacting happily or angrily. In the second experiment the stimuli were the same as in the first but participants were asked to ignore either the visual or the auditory information. The findings from both experiments indicated that when the reliability of the auditory cue was decreased, participants placed more weight on the visual cue in their emotional judgments. This in turn translated into increased emotion recognition accuracy for the multisensory condition. Those findings thus point to a common mechanism of multisensory integration of emotional signals, irrespective of social stimulus complexity. While the study by Piwek et al. (2015) is only one example of how the presented stimulus set can be used to examine audiovisual integration of emotional social signals, it demonstrates its versatility, flexibility and reliability.

Overall, the stimulus set developed is a simple, customisable and compact tool to explore both unimodal and multimodal aspects of emotional social interactions. We envision a broad range of applications in areas such as: social perception in typical and atypical developing individuals, detection of social signals and perception of social interactions using fMRI paradigm, multisensory integration of emotional and social signals, detection of emotional non-verbal cues, and many more.

## Electronic supplementary material

Below is the link to the electronic supplementary material.

(ZIP 270 MB)

(PDF 44.0 KB)

(PDF 53.1 KB)

(PDF 81.3 KB)

(PDF 85.9 KB)

## Footnotes

1 Due to problems with missing data points and corrupted audio files we had to exclude 4 emotional displays (two angry-medium-inquiry from couple ‘A’, one angry-low-deliberation from couple ‘C’ and one angry-medium-deliberation from couple ‘C’, and 10 neutral displays (one inquiry and one deliberation from couple ‘A’, and all four neutral from couple ‘D’ and ‘G’). See Supplementary Table 3 for details.
