# Supplementary material for: A dyadic stimulus set of audiovisual affective displays for the study of multisensory, emotional, social interactions
Source: Behav Res Methods. 2015 Nov 5;48(4):1285–95. doi: 10.3758/s13428-015-0654-4 (PMC5101291; doi:10.3758/s13428-015-0654-4)
Supplement: Supplementary file 2 — (PDF 44.0 KB) [file 13428_2015_654_MOESM2_ESM.pdf]

Supplementary Table 2: Anatomical location of markers for Plug-in Gait model.

Definition	Position on Patient
Left front head	Left temple
Right front head	Right temple
Left back head	Left back of head
Right back head	Right back of head
7th cervical vertebra	On the spinous process of the 7th cervical vertebra
10th thoracic vertebra	On the spinous process of the 10th thoracic vertebra
Clavicle	On the jugular notch where the clavicles meet the sternum
Sternum	On the xiphoid process of the sternum
Right back	Anywhere over the right scapula
Left ASIS	Left anterior superior iliac spine
Right ASIS	Right anterior superior iliac spine
Left PSI	Left posterior superior iliac spine
Right PSI	Right posterior superior iliac spine
Left shoulder	On the acromio-clavicular joint
Left upper arm	On the upper lateral 1/3 surface of the left arm
Left elbow	On the lateral epicondyle
Left forearm	On the lower lateral 1/3 surface of the left forearm
Left wrist marker A	At the thumb side on the posterior of the left wrist, close to the wrist joint
Left wrist marker B	At the little finger side on the posterior of the left wrist, close to the wrist joint
Left finger	Just proximal to the middle knuckle on the left hand
Right shoulder	On the acromio-clavicular joint
Right upper arm	On the lower lateral 1/3 surface of the right arm
Right elbow	On the lateral epicondyle approximating the elbow joint axis
Right forearm	On the lower lateral 1/3 surface of the right forearm
Right wrist marker A	At the thumb side of a bar on the posterior of the right wrist
Right wrist marker B	At the little finger side of a bar on the posterior of the right wrist
Right finger	Just proximal to the middle knuckle on the right hand.
Left thigh	Over the lower lateral 1/3 surface of the left thigh in line with the hip and knee joint centres
Left knee	On the flexion-extension axis of the left knee
Left tibia	Over the lower 1/3 surface of the left shank
Left ankle	On the lateral malleolus along an imaginary line that passes through the transmalleolar axis
Left heel	On the calcaneous at the same height above the plantar surface of the foot as the toe marker
Left toe	Over the second metatarsal head, on the mid-foot side of the equinus break between fore-foot and mid-foot
Right thigh	Over the lower lateral 1/3 surface of the right thigh
Right knee	On the flexion-extension axis of the right knee
Right tibia	Over the lower 1/3 surface of the right shank
Right ankle	On the lateral malleolus along an imaginary line that passes through the transmalleolar axis
Right heel	On the calcaneous at the same height above the plantar surface of the foot as the toe marker
Right toe	Over the second metatarsal head, on the mid-foot side of the equinus break between fore-foot and mid-foot
