# Supplementary material for: A dyadic stimulus set of audiovisual affective displays for the study of multisensory, emotional, social interactions
Source: Behav Res Methods. 2015 Nov 5;48(4):1285–95. doi: 10.3758/s13428-015-0654-4 (PMC5101291; doi:10.3758/s13428-015-0654-4)
Supplement: Supplementary file 3 — (PDF 53.1 KB) [file 13428_2015_654_MOESM3_ESM.pdf]

Supplementary Table 1: Scenarios given to actor during emotional interaction.

Emotion	Intensity	Scenario
Angry	Low	You've just discovered that youve been charged extra for one product that was clearly on promotional offer.
		You have been in rush for important meeting, and a sudden rain completely soaked you.
	Medium	Someone just stepped on your toe.
		Someone cut in line, when youve been waiting for over 20 minutes.
	High	You have just discovered that your wallet has been stolen.
		Your parents or partner just got into a big argument with you about something silly.
Happy	Low	You wake up on a Saturday after a number of wintry-cold rainy days, and the temperature is around 20°C.
		You have just finished this report youve been working on for two weeks.
	Medium	You unexpectedly run into someone you like very much and havent seen in a long time.
		You got an amazing bargain in the local retail store sale.
	High	You buy a lottery ticket and you win 10,000 pounds instantly.
		Its your birthday and your friends got you unexpected and awesome gift.
