# Supplementary material for: A dyadic stimulus set of audiovisual affective displays for the study of multisensory, emotional, social interactions
Source: Behav Res Methods. 2015 Nov 5;48(4):1285–95. doi: 10.3758/s13428-015-0654-4 (PMC5101291; doi:10.3758/s13428-015-0654-4)
Supplement: Supplementary file 4 — (PDF 81.3 KB) [file 13428_2015_654_MOESM4_ESM.pdf]

Supplementary Material 1: *R* routine for labelling column names for TXT coordinate files included in Supplementary Material 2. Each TXT coordinate file consist of 90 columns (45 columns for each actor). The naming system is as follows: actor number (a1,a2), 3D coordinate (x,y,z), specific limb (with l - left, r - right). Three commented numbers at the end indicate a column number.

```
#Read all columns of single coordinate file to 'data.frame' object
single_coordinate_file <- read.table("file_name.txt", sep="\t")

#Name each column
colnames(single_coordinate_file) <-
  c("a1.x.l_hip","a1.y.l_hip","a1.z.l_hip",#1,2,3
    "a1.x.r_hip","a1.y.r_hip","a1.z.r_hip",#4,5,6
    "a1.x.l_knee","a1.y.l_knee","a1.z.l_knee",#7,8,9
    "a1.x.r_knee","a1.y.r_knee","a1.z.r_knee",#10,11,12
    "a1.x.l_ankle","a1.y.l_ankle","a1.z.l_ankle",#13,14,15
    "a1.x.r_ankle","a1.y.r_ankle","a1.z.r_ankle",#16,17,18
    "a1.x.l_shoulder","a1.y.l_shoulder","a1.z.l_shoulder",#19,20,21
    "a1.x.r_shoulder","a1.y.r_shoulder","a1.z.r_shoulder",#22,23,24
    "a1.x.clavicle","a1.y.clavicle","a1.z_clavicle",#25,26,27
    "a1.x.head","a1.y.head","a1.z.head",#28,29,30
    "a1.x.l_elbow","a1.y.l_elbow","a1.z.l_elbow",#31,32,33
    "a1.x.r_elbow","a1.y.r_elbow","a1.z.r_elbow",#34,35,36
    "a1.x.l_hand","a1.y.l_hand","a1.z.l_hand",#37,38,39
    "a1.x.r_hand","a1.y.r_hand","a1.z.r_hand",#40,41,42
    "a1.x.pelvis","a1.y.pelvis","a1.z.pelvis",#43,44,45
    "a2.x.l_hip","a2.y.l_hip","a2.z.l_hip",#46,47,48
    "a2.x.r_hip","a2.y.r_hip","a2.z.r_hip",#49,50,51
    "a2.x.l_knee","a2.y.l_knee","a2.z.l_knee",#52,53,54
    "a2.x.r_knee","a2.y.r_knee","a2.z.r_knee",#55,56,57
    "a2.x.l_ankle","a2.y.l_ankle","a2.z.l_ankle",#58,59,60
    "a2.x.r_ankle","a2.y.r_ankle","a2.z.r_ankle",#61,62,63
    "a2.x.l_shoulder","a2.y.l_shoulder","a2.z.l_shoulder",#64,65,66
    "a2.x.r_shoulder","a2.y.r_shoulder","a2.z.r_shoulder",#67,68,69
    "a2.x.clavicle","a2.y.clavicle","a2.z_clavicle",#70,71,72
    "a2.x.head","a2.y.head","a2.z.head",#73,74,75
    "a2.x.l_elbow","a2.y.l_elbow","a2.z.l_elbow",#76,77,78
    "a2.x.r_elbow","a2.y.r_elbow","a2.z.r_elbow",#79,80,81
    "a2.x.l_hand","a2.y.l_hand","a2.z.l_hand",#82,83,84
    "a2.x.r_hand","a2.y.r_hand","a2.z.r_hand",#85,86,87
    "a2.x.pelvis","a2.y.pelvis","a2.z.pelvis")#88,89,90

#Save columns as a tab-delimited CSV file
write.csv(single_coordinate_file, "single_coordinate_file.csv",
        row.names = FALSE)
```
