# Supplementary material for: A dyadic stimulus set of audiovisual affective displays for the study of multisensory, emotional, social interactions
Source: Behav Res Methods. 2015 Nov 5;48(4):1285–95. doi: 10.3758/s13428-015-0654-4 (PMC5101291; doi:10.3758/s13428-015-0654-4)
Supplement: Supplementary file 5 — (PDF 85.9 KB) [file 13428_2015_654_MOESM5_ESM.pdf]

Supplementary Table 3: Characteristic of all 238 stimuli of the set with the outcomes of normative study. Top part of the table (above double horizontal line) shows the summary of all conditions (i.e. emotion, intensity, dialogues type with accuracy and condifence ratings) collapsed across all individual displays. The remaining table below double horizontal line shows results for all individual displays. Abbreviations stand for: file - name of display files as represented in the stimuli set database (each letter from file column represents a different couple), dial. - dialogue type (del. - delibration, enq. - enquiry), rep. - repetition, exp. - actors' experience (0 - non-experienced, 1 - experienced), dur. - display duration (seconds), accur. - average identification accuracy (in %), conf. - average confidence rating. Supplementary Material 2 (with stimuli set files) also includes a version of this Supplementary Table in *Microsoft Excel* XLS format for easier browsing.

	Display details						Audio-visual		Visual		Auditory	
file	emotion	intensity	dial.	rep.	exp.	dur.	acc.	conf.	acc.	conf.	acc.	conf.
	Angry	Low	del.				65	6	62	5	34	5
	Angry	Low	inq.				62	6	62	5	35	5
	Angry	Medium	del.				79	7	77	7	55	6
	Angry	Medium	inq.				76	6	76	6	53	6
	Angry	High	del.				85	8	86	8	75	7
	Angry	High	inq.				93	7	90	7	71	6
	Happy	Low	del.				85	6	84	6	71	6
	Happy	Low	inq.				80	6	77	6	78	5
	Happy	Medium	del.				88	7	89	7	66	6
	Happy	Medium	inq.				88	7	87	7	71	6
	Happy	High	del.				90	8	80	7	68	6
	Happy	High	inq.				90	8	78	7	68	6
A01	Happy	Low	inq.	1	0	3.00	53	5	73	4	75	5
A02	Happy	Low	inq.	2	0	3.00	73	6	80	5	83	6
A03	Happy	Medium	inq.	1	0	3.00	87	6	67	5	75	6
A04	Happy	Medium	inq.	2	0	3.00	87	6	73	5	92	6
A05	Happy	High	inq.	1	0	3.00	100	8	93	8	92	7
A06	Happy	High	inq.	2	0	3.00	87	7	80	7	83	6
A07	Angry	Low	inq.	1	0	3.16	40	6	13	4	58	5
A08	Angry	Low	inq.	2	0	3.66	67	6	13	4	67	5
A09	Angry	High	inq.	1	0	3.00	93	7	13	4	92	6
A10	Angry	High	inq.	2	0	3.66	100	7	67	5	92	7
A11	Happy	Low	del.	1	0	3.00	60	6	87	5	67	6
A12	Happy	Low	del.	2	0	3.00	80	6	80	6	67	6
A13	Happy	Medium	del.	1	0	3.00	100	7	93	6	100	7
A14	Happy	Medium	del.	2	0	3.00	80	6	73	6	83	6
A15	Happy	High	del.	1	0	3.00	93	7	73	6	100	7
A16	Happy	High	del.	2	0	3.00	100	8	80	6	100	7
A17	Angry	Low	del.	1	0	3.00	73	6	20	4	58	6
A18	Angry	Low	del.	2	0	3.00	80	6	27	6	67	5
A19	Angry	Medium	del.	1	0	3.00	80	5	27	5	75	5
A20	Angry	Medium	del.	2	0	3.00	100	6	27	4	92	6
A21	Angry	High	del.	1	0	3.00	93	8	47	5	100	9
A22	Angry	High	del.	2	0	3.66	100	8	53	6	92	8
A23	Neutral	NA	inq.	1	0	3.00	NA	4	NA	5	NA	5
A24	Neutral	NA	del.	2	0	3.00	NA	5	NA	5	NA	4
B01	Happy	Low	inq.	1	1	3.00	47	6	87	5	25	4
B02	Happy	Low	inq.	2	1	3.00	93	7	67	5	92	5
B03	Happy	Medium	inq.	1	1	3.00	100	7	73	6	92	7
B04	Happy	Medium	inq.	2	1	3.00	27	6	67	5	58	6
B05	Happy	High	inq.	1	1	4.00	20	6	80	6	33	5
B06	Happy	High	inq.	2	1	4.00	100	8	67	5	83	7
B07	Angry	Low	inq.	1	1	3.00	87	5	27	5	92	6
B08	Angry	Low	inq.	2	1	3.00	93	6	20	5	83	6
B09	Angry	Medium	inq.	1	1	3.00	87	6	33	4	83	6
B10	Angry	Medium	inq.	2	1	3.00	100	8	40	5	100	9

		Display details					Audio-visual		Visual		Auditory	
file	emotion	intensity	dial.	rep.	exp.	dur.	acc.	conf.	acc.	conf.	acc.	conf.
B11	Angry	High	inq.	1	1	3.00	93	8	80	6	100	9
B12	Angry	High	inq.	2	1	3.00	100	9	47	5	100	8
B13	Happy	Low	del.	1	1	3.33	93	6	80	5	92	6
B14	Happy	Low	del.	2	1	3.00	67	5	67	6	75	5
B15	Happy	Medium	del.	1	1	3.16	80	7	53	6	75	6
B16	Happy	Medium	del.	2	1	3.33	73	7	60	6	75	7
B17	Happy	High	del.	1	1	4.66	53	7	33	7	8	8
B18	Happy	High	del.	2	1	4.00	93	8	87	6	50	6
B19	Angry	Low	del.	1	1	3.00	67	6	13	5	58	5
B20	Angry	Low	del.	2	1	3.16	73	6	20	5	50	5
B21	Angry	Medium	del.	1	1	3.66	100	8	20	5	83	8
B22	Angry	Medium	del.	2	1	3.66	100	8	53	5	100	8
B23	Angry	High	del.	1	1	4.66	100	9	87	7	100	9
B24	Angry	High	del.	2	1	4.66	93	8	87	7	100	9
B25	Neutral	NA	inq.	1	1	3.00	NA	5	NA	4	NA	5
B26	Neutral	NA	inq.	2	1	3.00	NA	6	NA	4	NA	5
B27	Neutral	NA	del.	1	1	3.00	NA	5	NA	5	NA	5
B28	Neutral	NA	del.	2	1	3.00	NA	5	NA	5	NA	6
C01	Happy	Low	inq.	1	1	3.00	87	7	93	6	75	6
C02	Happy	Low	inq.	2	1	3.00	80	6	87	5	92	7
C03	Happy	Medium	inq.	1	1	3.00	93	8	93	6	92	8
C04	Happy	Medium	inq.	2	1	3.00	100	7	73	5	92	7
C05	Happy	High	inq.	1	1	3.00	100	8	80	6	92	8
C06	Happy	High	inq.	2	1	3.00	100	8	73	5	100	8
C07	Angry	Low	inq.	1	1	3.00	53	6	20	5	75	6
C08	Angry	Low	inq.	2	1	3.00	33	6	7	4	25	6
C09	Angry	Medium	inq.	1	1	3.00	87	6	13	5	83	7
C10	Angry	Medium	inq.	2	1	3.00	40	5	13	5	33	5
C11	Angry	High	inq.	1	1	3.00	87	7	47	5	58	7
C12	Angry	High	inq.	2	1	3.00	73	6	47	5	67	6
C13	Happy	Low	del.	1	1	3.00	80	7	87	5	92	7
C14	Happy	Low	del.	2	1	3.00	87	6	60	4	92	7
C15	Happy	Medium	del.	1	1	3.00	87	7	73	6	100	7
C16	Happy	Medium	del.	2	1	3.00	93	7	80	4	92	7
C17	Happy	High	del.	1	1	3.00	93	8	73	6	83	7
C18	Happy	High	del.	2	1	3.00	93	7	67	5	100	7
C19	Angry	Low	del.	1	1	3.00	33	6	20	5	42	6
C20	Angry	Medium	del.	1	1	3.00	47	6	47	6	42	6
C21	Angry	High	del.	1	1	3.00	87	7	73	6	75	8
C22	Angry	High	del.	2	1	3.00	27	7	47	5	33	7
C23	Neutral	NA	inq.	1	1	3.00	NA	5	NA	4	NA	5
C24	Neutral	NA	inq.	2	1	3.00	NA	6	NA	3	NA	6
C25	Neutral	NA	del.	1	1	3.00	NA	5	NA	5	NA	5
C26	Neutral	NA	del.	2	1	3.00	NA	6	NA	5	NA	6
D01	Angry	Low	inq.	1	0	3.00	47	6	67	6	67	5
D02	Angry	Low	inq.	2	0	3.00	40	5	73	6	50	5
D03	Angry	Medium	inq.	1	0	3.00	73	6	87	7	58	5
D04	Angry	Medium	inq.	2	0	3.00	33	6	60	5	50	5
D05	Angry	High	inq.	1	0	3.00	87	6	93	7	92	6
D06	Angry	High	inq.	2	0	2.50	87	7	87	7	75	6
D07	Happy	Low	inq.	1	0	3.00	100	6	67	6	83	6
D08	Happy	Low	inq.	2	0	3.00	100	8	100	6	100	7
D09	Happy	Medium	inq.	1	0	3.00	93	7	93	7	100	6
D10	Happy	Medium	inq.	2	0	3.00	80	7	87	6	67	6
D11	Happy	High	inq.	1	0	3.00	87	8	53	7	75	7
D12	Happy	High	inq.	2	0	3.00	100	8	87	6	83	7
D13	Angry	Low	del.	1	0	3.66	67	6	60	5	83	6
D14	Angry	Low	del.	2	0	3.16	73	6	60	6	67	5
D15	Angry	Medium	del.	1	0	3.00	80	6	73	6	92	7
D16	Angry	Medium	del.	2	0	3.00	60	6	87	8	42	5
D17	Angry	High	del.	1	0	3.33	80	7	93	8	92	7
D18	Angry	High	del.	2	0	3.33	73	5	87	8	67	5
D19	Happy	Low	del.	1	0	3.00	80	6	73	6	75	6
D20	Happy	Low	del.	2	0	3.00	100	7	80	6	92	6
D21	Happy	Medium	del.	1	0	3.00	87	7	60	6	92	6
D22	Happy	Medium	del.	2	0	3.66	80	7	80	6	75	6
D23	Happy	High	del.	1	0	4.00	100	8	80	7	100	8
D24	Happy	High	del.	2	0	3.00	87	7	87	7	75	6

		Display details					Audio-visual		Visual		Auditory	
file	emotion	intensity	dial.	rep.	exp.	dur.	acc.	conf.	acc.	conf.	acc.	conf.
E01	Happy	Low	inq.	1	0	3.00	87	6	73	6	58	6
E02	Happy	Low	inq.	2	0	3.00	27	6	53	5	50	5
E03	Happy	Medium	inq.	1	0	3.00	93	7	47	6	92	6
E04	Happy	Medium	inq.	2	0	3.00	73	6	53	6	75	6
E05	Happy	High	inq.	1	0	3.00	80	7	33	7	58	6
E06	Happy	High	inq.	2	0	3.00	80	7	47	6	50	6
E07	Angry	Low	inq.	1	0	3.00	53	5	67	5	33	6
E08	Angry	Low	inq.	2	0	3.00	67	6	33	5	58	5
E09	Angry	Medium	inq.	1	0	3.00	60	6	60	6	92	6
E10	Angry	Medium	inq.	2	0	3.00	87	7	80	6	58	6
E11	Angry	High	inq.	1	0	3.00	67	6	73	6	58	6
E12	Angry	High	inq.	2	0	3.00	100	7	60	6	100	7
E13	Happy	Low	del.	1	0	3.00	93	7	40	6	100	7
E14	Happy	Low	del.	2	0	3.00	60	6	53	6	58	5
E15	Happy	Medium	del.	1	0	3.00	80	8	33	5	92	8
E16	Happy	Medium	del.	2	0	3.00	87	7	47	5	100	6
E17	Happy	High	del.	1	0	3.00	87	7	27	6	83	7
E18	Happy	High	del.	2	0	3.00	87	8	40	6	92	8
E19	Angry	Low	del.	1	0	3.00	60	6	40	5	58	5
E20	Angry	Low	del.	2	0	3.00	67	6	20	6	75	5
E21	Angry	Medium	del.	1	0	3.00	80	7	60	6	83	6
E22	Angry	Medium	del.	2	0	3.00	67	7	67	5	67	6
E23	Angry	High	del.	1	0	3.00	100	8	60	7	92	8
E24	Angry	High	del.	2	0	3.00	93	6	60	5	75	6
E25	Neutral	NA	inq.	1	0	3.00	NA	6	NA	5	NA	5
E26	Neutral	NA	inq.	2	0	3.00	NA	4	NA	5	NA	6
E27	Neutral	NA	inq.	1	0	3.00	NA	5	NA	4	NA	5
E28	Neutral	NA	inq.	2	0	3.00	NA	5	NA	4	NA	5
F01	Happy	Low	inq.	1	0	3.00	87	5	87	5	58	5
F02	Happy	Low	inq.	2	0	3.00	93	6	93	6	83	6
F03	Happy	Medium	inq.	1	0	3.00	100	7	67	5	83	7
F04	Happy	Medium	inq.	2	0	2.66	93	7	73	7	100	7
F05	Happy	High	inq.	1	0	2.66	100	8	87	7	92	6
F06	Happy	High	inq.	2	0	3.16	100	8	80	6	75	8
F07	Angry	Low	inq.	1	0	3.00	47	5	47	5	33	4
F08	Angry	Low	inq.	2	0	3.00	33	5	13	5	25	6
F09	Angry	Medium	inq.	1	0	3.00	53	5	73	5	42	6
F10	Angry	Medium	inq.	2	0	3.00	60	5	60	6	83	5
F11	Angry	High	inq.	1	0	3.00	93	7	87	8	100	6
F12	Angry	High	inq.	2	0	2.50	93	7	100	7	92	8
F13	Happy	Low	del.	1	0	3.00	93	7	67	6	100	7
F14	Happy	Low	del.	2	0	3.00	93	5	87	6	83	6
F15	Happy	Medium	del.	1	0	3.00	100	7	100	7	92	7
F16	Happy	Medium	del.	2	0	3.00	100	7	73	5	100	7
F17	Happy	High	del.	1	0	3.00	100	8	73	7	92	7
F18	Happy	High	del.	2	0	3.00	87	7	73	7	92	6
F19	Angry	Low	del.	1	0	3.00	20	7	33	7	25	6
F20	Angry	Low	del.	2	0	3.00	13	5	33	4	17	6
F21	Angry	Medium	del.	1	0	3.00	40	5	47	5	50	5
F22	Angry	Medium	del.	2	0	3.00	40	6	73	7	0	6
F23	Angry	High	del.	1	0	3.00	33	6	73	6	42	6
F24	Angry	High	del.	2	0	3.00	73	6	73	6	83	6
F25	Neutral	NA	inq.	1	0	3.00	NA	5	NA	5	NA	5
F26	Neutral	NA	inq.	2	0	2.83	NA	5	NA	6	NA	6
F27	Neutral	NA	inq.	1	0	3.00	NA	4	NA	5	NA	4
F28	Neutral	NA	inq.	2	0	3.00	NA	4	NA	5	NA	5
G01	Happy	Low	inq.	1	0	3.00	100	7	87	6	92	6
G02	Happy	Low	inq.	2	0	3.00	60	6	93	5	67	6
G03	Happy	Medium	inq.	1	0	2.66	93	7	87	6	83	8
G04	Happy	Medium	inq.	2	0	2.66	80	6	93	6	75	6
G05	Happy	High	inq.	1	0	2.66	100	8	80	6	100	7
G06	Happy	High	inq.	2	0	2.66	93	8	67	5	92	6
G07	Angry	Low	inq.	1	0	2.66	93	6	53	5	92	6
G08	Angry	Low	inq.	2	0	2.33	93	8	40	4	92	7
G09	Angry	Medium	inq.	1	0	2.66	100	8	33	6	100	8
G10	Angry	Medium	inq.	2	0	2.66	100	7	80	5	92	7

		Display details					Audio-visual		Visual		Auditory	
file	emotion	intensity	dial.	rep.	exp.	dur.	acc.	conf.	acc.	conf.	acc.	conf.
G11	Angry	High	inq.	1	0	2.33	100	9	80	7	92	8
G12	Angry	High	inq.	2	0	2.66	100	7	87	8	100	8
G13	Happy	Low	del.	1	0	2.66	100	7	73	6	100	7
G14	Happy	Low	del.	2	0	2.33	100	7	80	5	100	8
G15	Happy	Medium	del.	1	0	3.00	93	7	87	7	92	6
G16	Happy	Medium	del.	2	0	2.50	100	8	87	5	100	7
G17	Happy	High	del.	1	0	3.00	93	8	80	7	92	6
G18	Happy	High	del.	2	0	3.00	100	8	100	6	100	7
G19	Angry	Low	del.	1	0	3.00	87	6	60	7	100	6
G20	Angry	Low	del.	2	0	3.00	100	6	27	6	83	5
G21	Angry	Medium	del.	1	0	3.00	87	7	87	7	100	7
G22	Angry	Medium	del.	2	0	3.50	100	8	67	5	100	9
G23	Angry	High	del.	1	0	3.03	93	8	80	9	100	9
G24	Angry	High	del.	2	0	3.00	93	8	93	6	100	8
H01	Happy	Low	inq.	1	1	3.00	87	6	73	5	83	7
H02	Happy	Low	inq.	2	1	3.00	93	7	93	5	83	7
H03	Happy	Medium	inq.	1	1	3.00	100	8	73	6	100	9
H04	Happy	Medium	inq.	2	1	3.00	93	7	87	6	100	8
H05	Happy	High	inq.	1	1	2.66	93	9	60	6	100	9
H06	Happy	High	inq.	2	1	3.03	87	8	27	7	75	7
H07	Angry	Low	inq.	1	1	3.00	47	5	0	5	42	4
H08	Angry	Low	inq.	2	1	3.00	60	5	20	5	75	5
H09	Angry	Medium	inq.	1	1	3.00	87	6	20	5	83	6
H10	Angry	Medium	inq.	2	1	3.00	53	6	40	5	75	5
H11	Angry	High	inq.	1	1	3.00	100	8	80	7	100	7
H12	Angry	High	inq.	2	1	3.00	100	8	47	6	100	8
H13	Happy	Low	del.	1	1	3.00	100	8	73	5	92	8
H14	Happy	Low	del.	2	1	3.00	87	5	100	6	42	5
H15	Happy	Medium	del.	1	1	3.50	100	8	87	6	100	8
H16	Happy	Medium	del.	2	1	3.00	93	7	67	7	92	6
H17	Happy	High	del.	1	1	3.00	100	8	93	7	83	8
H18	Happy	High	del.	2	1	3.50	67	7	33	6	8	6
H19	Angry	Low	del.	1	1	3.00	53	4	27	5	42	4
H20	Angry	Low	del.	2	1	3.00	73	5	13	5	58	5
H21	Angry	Medium	del.	1	1	3.00	93	7	13	6	92	6
H22	Angry	Medium	del.	2	1	3.00	87	5	13	6	92	6
H23	Angry	High	del.	1	1	3.50	93	8	67	6	100	8
H24	Angry	High	del.	2	1	3.66	100	9	87	6	100	9
H25	Neutral	NA	inq.	1	1	2.66	NA	5	NA	3	NA	6
H26	Neutral	NA	inq.	2	1	2.50	NA	5	NA	4	NA	4
H27	Neutral	NA	del.	1	1	2.66	NA	4	NA	4	NA	5
H28	Neutral	NA	del.	2	1	2.50	NA	4	NA	5	NA	5
I01	Happy	Low	inq.	1	1	3.00	87	6	60	5	92	6
I02	Happy	Low	inq.	2	1	3.00	93	7	47	5	92	7
I03	Happy	Medium	inq.	1	1	3.00	100	8	27	6	92	8
I04	Happy	Medium	inq.	2	1	3.00	100	8	47	5	92	8
I05	Happy	High	inq.	1	1	3.33	93	8	87	7	42	6
I06	Happy	High	inq.	2	1	3.00	100	8	40	6	83	7
I07	Angry	Low	inq.	1	1	3.00	87	6	47	5	67	6
I08	Angry	Low	inq.	2	1	3.00	73	6	67	5	75	5
I09	Angry	Medium	inq.	1	1	3.00	93	8	87	7	92	7
I10	Angry	Medium	inq.	2	1	3.00	100	8	73	7	92	8
I11	Angry	High	inq.	1	1	3.00	100	8	93	9	100	8
I12	Angry	High	inq.	2	1	3.00	100	9	87	7	100	9
I13	Happy	Low	del.	1	1	3.00	87	8	47	6	92	7
I14	Happy	Low	del.	2	1	3.00	73	6	40	6	92	6
I15	Happy	Medium	del.	1	1	3.00	87	8	20	6	83	8
I16	Happy	Medium	del.	2	1	3.00	73	7	7	7	67	7
I17	Happy	High	del.	1	1	3.00	100	9	67	7	100	8
I18	Happy	High	del.	2	1	3.00	87	8	53	5	75	7
I19	Angry	Low	del.	1	1	3.00	80	6	53	5	75	6
I20	Angry	Low	del.	2	1	3.00	93	7	60	6	100	7
I21	Angry	Medium	del.	1	1	3.66	93	8	87	7	100	7
I22	Angry	Medium	del.	2	1	3.00	93	9	93	8	100	8
I23	Angry	High	del.	1	1	3.33	100	9	87	9	100	8
I24	Angry	High	del.	2	1	3.00	100	9	93	9	100	9
I25	Neutral	NA	inq.	1	1	3.00	NA	5	NA	5	NA	6
I26	Neutral	NA	inq.	2	1	2.66	NA	7	NA	4	NA	7
I27	Neutral	NA	del.	1	1	3.00	NA	5	NA	5	NA	5
I28	Neutral	NA	del.	2	1	3.00	NA	6	NA	5	NA	6
